# Remnant preservation may improve proprioception after anterior cruciate ligament reconstruction

**DOI:** 10.1186/s10195-022-00641-y

**Published:** 2022-04-27

**Authors:** Eunshinae Cho, Jiebo Chen, Caiqi Xu, Jinzhong Zhao

**Affiliations:** Department of Sports Medicine, Shanghai Jiao Tong University Affiliated Sixth People’s Hospital, 600 Yishan Road, Shanghai, 200233 China

**Keywords:** ACL, Proprioception, Remnant, JPS

## Abstract

**Aim:**

Our aim was to evaluate the literature investigating proprioception improvement after anterior cruciate ligament reconstruction (ACLR) and test the hypothesis that ACL tibial remnant-preserving reconstruction (ACLR-R) is more beneficial than standard technique (ACLR-S) in terms of postoperative proprioceptive function with various reported tests, including joint position sense (JPS) and threshold to detect passive motion (TTDPM).

**Methods:**

An online search was performed in Embase, MEDLINE/PubMed, Cochrane, SPORTDiscus, and Web of Science databases before 5 October 2020, on the basis of the guidelines of the Preferred Reporting Items for Systematic reviews and Meta-Analyses (PRISMA) statement. Key terms [(‘ACLR’ or ‘ACL-R’ or ‘anterior cruciate ligament reconstruction’) AND (‘remnant’ or ‘stump’) AND (‘proprioception’ or ‘proprioceptive’)] were used. The Oxford Centre for Evidence-Based Medicine and The McMaster Critical Review Form for Quantitative Studies were used for quality assessment. In total, four articles comparing proprioceptive functions between ACLR-R and ACLR-S were included, two of which were randomized clinical trials rated as level of evidence II, and two were retrospective cohort studies rated as level of evidence III. The outcomes were then compared. Evaluation of proprioception involved joint position sense (JPS) [reproduction of active positioning (RAP) and reproduction of passive positioning (RPP)] and threshold to detect passive motion (TTDPM) tests.

**Results:**

Only four studies were included, with a total of 234 patients (119 ACLR-R patients and 115 ACLR-S patients). High heterogeneity in characteristics and outcome measurements was observed among the studies. Three studies performed sparing technique, and one performed tensioning technique. One study tested RAP and reported better results at an average of 7 months follow-up in ACLR-R (*P* < 0.05). Three studies tested RPP, one of which measured RPP within 12 months after surgery and reported better results in ACLR-R than in ACLR-S (*P* < 0.05). The other two studies reported similar results; however, the findings of one study were statistically insignificant. TTDPM was tested in one study, with no statistically significant difference found.

**Conclusion:**

The current literature, although limited, reported proprioception improvement after ACLR-R (compared with ACLR-S) in terms of JPS. However, owing to the heterogeneity of the relevant studies, further research is required to determine remnant preservation effect on knee proprioceptive restoration.

**Level of evidence:**

Level III, systematic review of Level II and III studies.

## Introduction

Proprioception, a sensory modality responsible for the sensation of joint movement and position, plays a crucial role in the afferent–efferent neuromuscular control arc and normal joint performance [26, 32, 41, 42].

Proprioceptors, including Ruffini endings, Pacinian corpuscles, and Golgi tendon organs, are located at the tibial bone insertion area of the anterior cruciate ligament (ACL) [1, 9].

Therefore, ACL injury can cause damage and loss of proprioceptive receptors (based on the time between injury and surgery [14]) and can translate into a decrease in afferent information input [15, 16], leading to mechanical instability [22, 41]. Such decreased proprioception can adversely affect the dynamic stability of the knee, strength, and balance and can increase the risk of secondary ACL injury by 30–40 times [6, 18, 49].

In addition to the role of proprioceptive rehabilitation programs [32, 36], remnant-preserving ACL reconstruction (ACLR) is potentially beneficial for proprioception restoration, based on histological findings of the presence of proprioceptors in injured ACL 3 years after injury [17]. Owing to the majority of ACL tears occurring at the femoral insertion, the tibial attachment remains relatively intact in the anatomic position [21]. Studies suggest a potential benefit of preserving the tibial remnant during ACLR in the retention of proprioceptors [21, 25]. However, the advantage of remnant preservation is shown mainly in basic histological studies rather than in clinical studies [25].

Various studies have reported that ACL remnant preservation facilitates recovery of stability, enhances tissue healing, and even decreases graft rupture after surgery [4, 12, 24, 38, 46]. However, the relationship between functional stability and proprioception was overlooked in such investigations [18]. The presence of proprioceptors in the ACL remnant [17] informs surgeons to consider remnant-preserving ACLR for better knee joint proprioceptive recovery, which raises the question of whether histological findings and benefits could be translated into clinical proprioceptive improvements.

Therefore, the purpose of this review was to evaluate the current literature and research work focusing on proprioception improvement after ACLR. In addition, since most reviews focused on clinical aspects of improvement rather than proprioception, we also aimed to determine whether ACL tibial remnant-preserving reconstruction (ACLR-R) is more beneficial than standard technique (ACLR-S) in terms of postoperative proprioceptive function with various reported tests. We hypothesized that proprioception functions are better in the ACL remnant-preserving technique than in standard reconstruction techniques, regardless of the methods of proprioceptive measurement.

## Methods

### Search strategy

A literature search was performed on the basis of the guidelines of the Preferred Reporting Items for Systematic reviews and Meta-Analyses (PRISMA) statement [43]. Online databases (Embase, MEDLINE/PubMed, Cochrane, SPORTDiscus, and Web of Science) were searched for all English-language studies before 5 October 2020. Two reviewers separately searched these databases using key terms [(‘ACLR’ or ‘ACL-R’ or ‘anterior cruciate ligament reconstruction’) AND (‘remnant’ or ‘stump’) AND (‘proprioception’ or ‘proprioceptive’)]. References of the screened articles were also retrieved for potential inclusion. The authors of the studies were contacted for further clarifications when necessary.

### Inclusion and exclusion criteria

#### Participants

Human unilaterally surgical ACL-reconstructed participants without other knee ligament interventions or repair of ACL avulsion fractures were included in this study. We excluded studies on patients with systematic disorders (e.g., cardiac vascular or neurological disorders) and congenital deformities of the lower extremities as well as those on animals and cadavers.

#### Interventions and comparisons

ACLR with remnant preservation was considered as the experimental intervention in this systematic review. Double-bundle and single-bundle ACLRs with different sources of grafts were included. The control intervention was non-remnant standard ACLR. Studies comparing the various amounts of remnants but not with the debridement procedure were excluded, while studies comparing the remnants with debridement were included if separate evaluations of each group were available.

#### Outcome measurements

In total, the outcome measurements included: (1) proprioceptive evaluation involving joint position sense (JPS) [13] [reproduction of active positioning (RAP) and reproduction of passive positioning (RPP)] and threshold to detect passive motion (TTDPM) tests [8]; (2) balance or postural control tests; (3) objective knee stability examinations such as the anterior drawer test, Lachman test, KT-arthrometer measurement, and pivot-shift test; (4) patient-reported outcomes, including the International Knee Documentation Committee (IKDC) score, Tegner activity scale, Lysholm score, and Hospital for Special Surgery (HSS) score.

#### Study type

In this study, we included all published English-language randomized controlled trials, prospective cohort studies without randomization, and retrospective cohort studies or case series with historical controls (evidence levels I, II, III, IV) that reported the proprioceptive outcomes of ACLR-R versus ACLR-S [51]. No minimum follow-up period was employed. Case series without controls, case reports, and expert opinions (level IV or V) [51] as well as studies solely depicting functional outcomes without further proprioceptive outcomes, meeting abstracts, trial protocols, and systematic reviews were excluded.

### Data extraction

After the removal of duplicates, two independent reviewers screened the titles and abstracts of the studies for potential eligibility. Studies were further analyzed in full text if the abstract did not provide enough data to make a decision. A senior author was consulted when there were disagreements between the reviewers.

Data included: (1) general information (first author, publication year, country where study was performed, sample size, mean age, sex, mean time from injury to surgery, mean follow-up time, injury side, level of evidence, and study design); (2) ACLR surgical characteristics (ACL tear pattern, associated injury, remnant volume, number of bundles, graft diameter and type, surgical technique, tibial remnant management, complications, and rehabilitation); and (3) outcome measurements (proprioception assessment, balance or postural control tests, knee laxity and function, and patient-reported outcomes).

Studies that used ACL augmentation with selective ACL anteromedial or posterolateral bundle reconstruction were excluded from the review.

### Quality assessment

To evaluate the methodological quality of the evidence, a critical appraisal of all included studies was performed. The level of evidence was assessed according to the Oxford Centre for Evidence-Based Medicine [51]. The McMaster Critical Review Form for Quantitative Studies [27] was used to rate the methodological quality by evaluating the risk of bias within studies. This form consists of nine categories: citation, study purpose, literature, design, sample, outcomes, intervention, results, and conclusions and implications. Responses are marked as yes (1 point), no or not addressed (0 point), or not applicable (item does not count). The sum of the outcomes (0–15 points) divided by the sum of the applicable items represents the overall quality of the study assessed.

The independent reviewers examined the studies, and any discrepancy was resolved through discussions with the senior author. Kappa values were also calculated to assess the inter-rater agreement of each individual item.

### Data analysis

The results of the review are presented as a synthesis with the extracted data descriptively reported as medians (minimum–maximum) and means (standard deviation and/or minimum–maximum) for continuous variables and percentages for categorical data. Completing a meta-analysis or quantitative analysis was not feasible owing to several reasons, including the heterogeneity of ACL tear patterns (partial or complete or not reported), ACL remnant and graft statuses, remnant management techniques (remnant tensioning or sparing), application of different proprioceptive evaluation methods, and testing conditions in included studies. All basic data and clinical results were qualitatively compared and summarized in this review.

## Results

### Search results

**Fig. 1:**
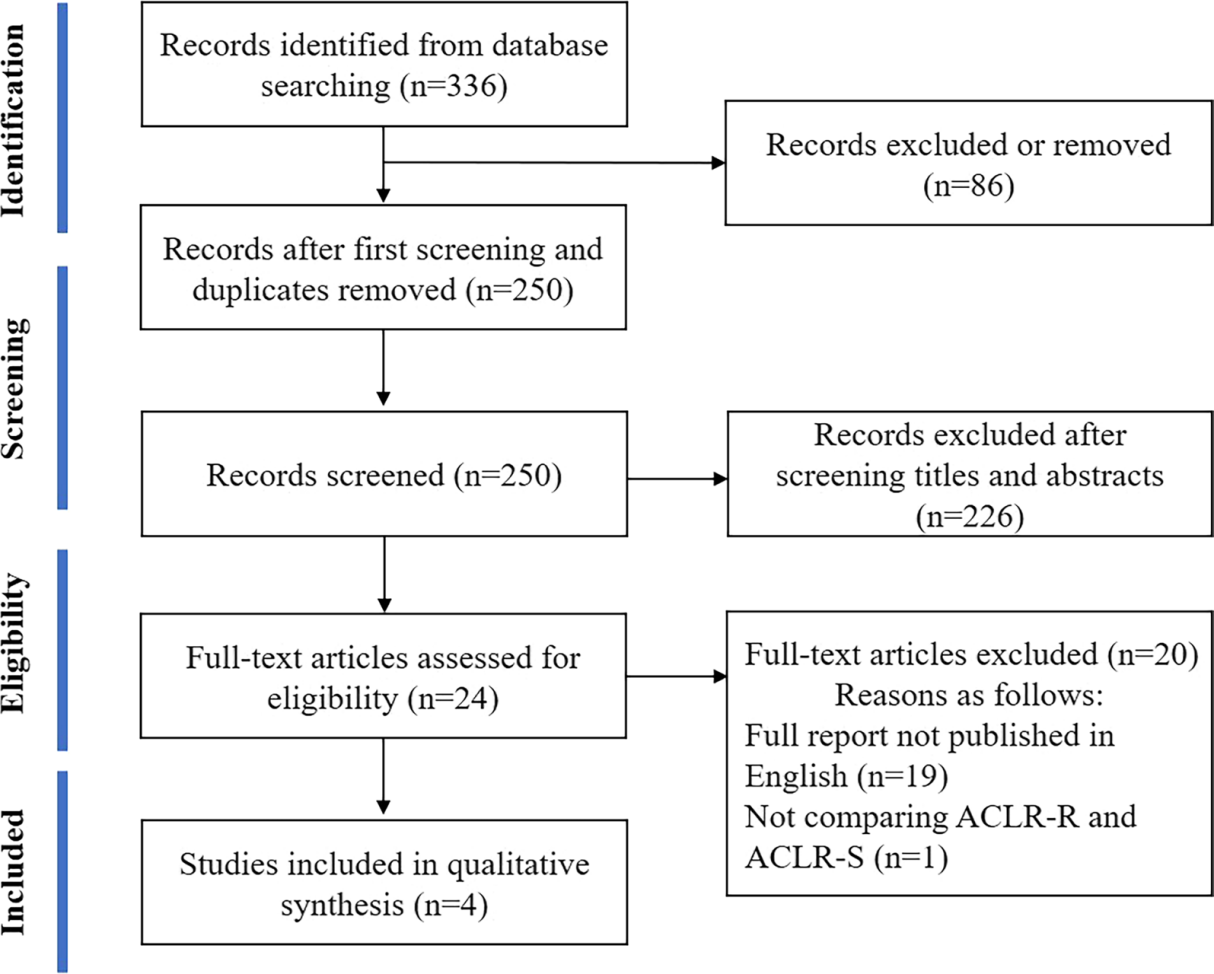
A flow diagram showing the PRISMA study selection of publications. ACLR-R, anterior cruciate ligament tibial remnant-preserving reconstruction; ACLR-S, anterior cruciate ligament standard reconstruction

A total of 336 studies were selected after reviewing the literature: 56 from Embase, 47 from MEDLINE/PubMed, 11 trials from the Cochrane library, 131 from SPORTDiscus, and 91 from Web of Science. After the first screening (duplication removal, eligibility criteria, and title-based exclusion), 250 studies were selected. Of these, 226 studies were excluded on the basis of the abstracts and exclusion criteria, and subsequently, 20 studies were excluded after full-text review. Finally, four studies [5, 11, 19, 28] were identified for qualitative synthesis (Fig. 1). In total, 234 participants (154 males, 80 females) were included in the four selected studies, with 119 participants who underwent ACLR-R and 115 participants who underwent ACLR-S.

### Critical appraisal in included studies

Of the four studies, two [5, 19] were randomized clinical trials rated as level of evidence II, while the remaining studies [11, 28] were retrospective cohort studies rated as level of evidence III (Table 1). None of the studies met all the McMaster critical appraisal criteria. Only one study justified the sample size with a power calculation [19]. Unbiased group assignment was conducted in only two studies [5, 19]. Cointervention bias was adequately addressed in all included studies, and the selected studies also provided information about the inclusion criteria and rehabilitation protocol [5, 11, 19, 28] (Table 2).

**Table 1:** Study characteristics

First author	Year	Country	Sample size (S/R)	Mean age (years) (S/R)	Sex (M/F)		Mean time from injury to reconstruction (months)		Mean follow-up (months)		Injury side (left/right)		Level of evidence	Study design
					S	R	S	R	S	R	S	R		
Hong et al. [16]	2012	China	45/45	28 (15–50)/34 (18–48)^a^	34/11	33/12	9.4 ± 25.9	10.3 ± 33.7	25.5 ± 2.4	25.8 ± 2.1	22/23	20/25	II	RCT
Andonovski et al. [5]	2017	Republic of Macedonia	33/33	28 (16–50)	58/8		NR		7 (6–8)		NR		II	RCT
Chen et al. [10]	2019	China	15/15	27.6 (16–44)/28.4 (17–48)	9/6	8/7	NR		20 (16–24)		8/7	10/5	III	Retrospective cohort study
Lee et al. [25]	2020	South Korea	22/26	30.0 ± 11.0/31.4 ± 10.2	7/19	5/17	NR (< 6)		25.5 (24–36)		NR		III	Retrospective cohort study

^a^Median (range)

NR, not reported; R, remnant preservation technique; RCT, randomized control trial; S, standard reconstruction technique

**Table 2:** Critical appraisal score for assessment of included studies

Assessment domain	Acceptable	Kappa value	Study			
			Hong et al. [16]	Andonovski et al. [5]	Chen et al. [10]	Lee et al. [25]
Study purpose						
Study purpose clearly stated	Yes (usually stated briefly in the abstract of the article, and again in more detail in the introduction applied to occupational therapy and/or the research question)	1.000	1	1	1	1
Literature						
Relevant background literature reviewed	Yes (providing a synthesis of relevant information such as previous work/research, and discussion of the clinical importance of the topic, justifying the need for the study being reported)	1.000	1	1	1	1
Study design						
Appropriateness of the design chosen for the study question	Yes	0.889	1	1	0	0
Sample						
Described in detail	Yes (who; characteristics; how many; how was sampling done; if more than one group, describing similarity between the groups)	1.000	1	0	1	1
Justified	Yes (sample size calculation; ethics procedures or informed consent obtained)	1.000	1	0	0	0
Outcomes						
Outcome measure reliably reported	Yes (test–retest reliability; inter-rater reliability)	1.000	–	–	–	–
Outcome measure validly reported	Yes (content validity; criterion validity)	0.985	1	–	–	1
Intervention						
Described in detail	Yes (focus, who delivered it, how often, setting)	1.000	1	1	1	1
Contamination avoided	Yes	0.984	1	1	N/A	N/A
Cointervention avoided	Yes	1.000	1	1	1	1
Results						
Reported in terms of statistical significance	Yes	1.000	1	1	1	1
Appropriate analysis methods	Yes	1.000	1	1	1	1
Clinical importance reported	Yes	1.000	1	1	1	1
Dropouts reported	Yes (reasons and how the analysis of the findings was handled, if applicable)	1.000	1	N/A	N/A	0
Conclusions and clinical implications						
Appropriate conclusion and relevant clinical influence	Yes	1.000	1	1	1	1
Overall CA score (range 0–15)/ Applicable CA items (range 0–15)			14/15	10/14	9/13	10/14
%			93	71	69	71

CA, critical appraisal; R, remnant preservation technique; S, standard reconstruction technique

Critical appraisal items were rated as yes (1), no (0), not addressed (–), or not applicable (N/A)

### Surgical characteristics

#### ACL injury and remnant and graft status

ACL remnant characteristics were described in all four studies [5, 11, 19, 28]. Only one of the studies reported the minimum remnant length to be quantitatively more than 20% of the native ACL [28]. Two of the studies reported a remnant diameter of over one-third [5] or half [19] of the original ACL diameter. Single-bundle ACLR was performed in all included studies [5, 11, 19, 28]. Two studies [5, 28] used an autologous hamstring graft, and the other studies [11, 19] used an allograft for reconstruction. The graft diameter ranged from 7 to 9 mm [11, 19, 28]. Only one study [5] did not describe the specific graft diameter (Table 3).

**Table 3:** Surgical characteristics of included studies

First author	ACL tear pattern	Associated injury, *n* (S/R)				Remnant	No. of bundles	Graft diameter, mm	Graft type	Surgical technique (S/R)		Tibial remnant management (S/R)	Complications	Rehabilitation (timing for partial/full weight-bearing after surgery, weeks)
		Medial meniscus injury	Lateral meniscus injury	Both meniscus injury	MCL injury									
										Femoral tunnel	Tibial tunnel			
Hong et al. [16]	Complete ACL knee injury	14/18	11/16	NR	4/2	Tibial insertion of the ACL remnant was intact and could be pulled to reach the femoral ACL insertion; the remnant diameter was more than half of the native ACL	Single	8–9	Allogeneic tibialis anterior or hamstring	AM portal	The center of the ACL remnant footprint	Removal/tensioning	Cyclops lesion formation (*n* = 3)	4/6
Andonovski et al. [5]	Partial or complete ACL rupture	28	11	4	NR	Residual remnant from the torn ACL has continuous ligament fibers down to the tibia and above to the wall of the intercondylar notch or to the posterior cruciate ligament; the remnant diameter was greater than one-third that of normal ACL	Single	NR	Autologous hamstring	AM or AAM portal	Middle of the remnant attachment/anteromedial or posterolateral to the remnant	Removal/sparing	NR	NR
Chen et al. [10]	Unspecified	NR				Retention of about 1 cm of fiber at the ligament tibial endpoint	Single	8–9	Allograft	AM portal	The center of the original ACL tibial stump	Removal/sparing and retention of about 1 cm of fiber at the tibial insertion	None	5/12
Lee et al. [25]	Unspecified	6/5	10/13	3/4	NR	ACL remnant of more than 7 mm (approximately 20% of the mean length of the normal ACL) in the remnant preservation group, while less than 7 mm in the remnant removal group	Single	7 or 8	Autologous hamstring	Outside-in	The center of the remaining ACL footprint	Removal/sparing	NR	2/6

AAM, accessory anteromedial; AM, anteromedial; ACL, anterior cruciate ligament; MCL, medial collateral ligament; NR, not reported; R, remnant preservation technique; S, standard reconstruction technique

#### Surgical techniques and remnant management

The hypothesis of this study was that ACL tibial remnant-preserving reconstruction (ACLR-R) is more beneficial than standard technique (ACLR-S) in terms of postoperative proprioceptive function with various reported tests, including joint position sense (JPS) and threshold to detect passive motion (TTDPM). Therefore, the remnant preservation and resection referred solely to the tibial side rather than both the femoral and tibial sides. The femoral tunnel placement method was reported in all studies [5, 11, 19, 28]. The tunnels were created via the anteromedial (AM) or accessory anteromedial (AAM) portal in three studies [5, 11, 19], and via the outside-in technique [3] in one study [28]. Furthermore, the tibial tunnel was positioned in the center of the ACL remnant footprint [11, 19, 28] in both ACLR-R and ACLR-S, except in one study [5], which positioned the tip of the tibial tunnel guide anteromedial or posterolateral to the ACL posterolateral or anteromedial residual bundle for ACLR-R to prevent damage of the residual bundle. The tibial remnant was removed in all ACLR-S patients. In ACLR-R, three of four studies [5, 11, 28] performed the sparing technique described by Lee et al. [30], while only one study [19] used the tensioning technique described by Ahn et al.[2] (Table 3).

#### Complication

Only two studies described complications after ACLR [11, 19]. Hong et al. [19] reported cyclops lesion formation (*n* = 3/55) during second-look arthroscopic evaluation and subsequently resected it. Chen et al. [11] found no complications such as bone tunnel enlargement, impingement, or cyclops lesion. No significant difference was reported between the ACLR-R and ACLR-S groups (Table 3).

#### Rehabilitation

Three studies [11, 19, 28] reported weight-bearing rehabilitation after ACLR. Partial weight-bearing and full weight-bearing exercises started at least 2 weeks and 6 weeks after reconstruction, respectively (Table 3). Rehabilitation protocols were identical for treatments and controls in all studies that provided such details.

### Outcomes

#### Proprioception assessment

##### JPS test-RPP

Three of four studies [11, 19, 28] measured RPP at different follow-up times. Chen et al. [11] analyzed RPP test results at 3, 6, and 12 months after surgery and found that the ACLR-R group had significantly better RPP results than ACLR-S in all testing conditions (knee flexion of 15°, 30°, and 45°; *P* < 0.05). Two studies [19, 28] followed up on participants for more than 24 months (24–36 months). One of the two studies that used the sparing technique reported a statistically significant difference in RPP test, indicating better proprioception in ACLR-S (knee flexion of 15° and 30°; *P* = 0.40 and *P* = 0.010). The other study [19] analyzed RPP test results at 3, 6, 9, 12, 18, and 24 months, but presented statistically insignificant findings (*P* = 0.739) (Table 4). Interestingly, both studies reporting JPS-RPP improvement were observational studies, while the one study reporting no significant difference was a clinical trial.

**Table 4:** Results of proprioception per included study: joint position sense

First author	Test mode (°/s)	Direction (°)	JPS ACLR-S (°)		JPS ACLR-R (°)		*P* value	Measured knee for JPS	JPS measured times	JPS recorded value	Balance or postural control tests	Physical examination	Patient-reported outcomes	Proprioception outcome measurements	Eye shades/earplugs
Hong et al. [16]	RPP (5)	90–15 extension	3.9 ± 2.2		3.6 ± 1.8		0.739 Mann–Whitney *U* test	Reconstructed knee	3	(Test angle − setting angle) × 3, mean value	NR	Lachman test Pivot-shift test Laxity—KT-1000 max force in 30° flexion	Lysholm IKDC	Biodex	√
Andonovski et al. [5]	RAP	90 extension	Before surgery 1.8 ± 0.78 after surgery 1.3 ± 0.97		Before surgery 1.5 ± 0.96 after surgery 0.5 ± 0.53		< 0.05	Reconstructed and healthy knee	3	(Test angle − setting angle) × 3, average value, involved knee – normal knee	NR	Side to side—Rolimeter in 20° and 90° of knee flexion (*P* < 0.0001)	NR	Biodex System 4 Pro	NR
Chen et al. [10]	RPP	Maximum extension between 0, 30, and 45		3, 6, 12 months after surgery		3, 6, 12 months after surgery	< 0.05	Reconstructed knee	5	(Test angle − setting angle) × 5, mean value	NR	Lachman test Anterior drawer test	Lysholm (*P* < 0.05) Tegner (*P* < 0.05)	NR	NR
			0°	3.52 ± 0.88/3.27 ± 0.92/3.15 ± 0.68	0°	2.13 ± 0.49/2.06 ± 0.74/2.02 ± 0.48									
			30°	5.84 ± 1.23/4.87 ± 1.02/4.26 ± 0.65	30°	3.43 ± 0.85/2.47 ± 0.66/2.55 ± 0.51									
			45°	6.78 ± 1.35/4.98 ± 1.46/4.52 ± 0.77	45°										
						3.52 ± 0.72/3.01 ± 0.91/2.84 ± 0.63									
Lee et al. [25]	RPP	Flexion between 15, 30, and 45	15°	1.36 ± 1.04°	15°	0.69 ± 0.56°	0.040 Mann–Whitney *U* test	Reconstructed and healthy knee	5	(Test angle − setting angle) × 5, mean value	One-leg hop test (*P* < 0.05) Single-limb standing (*P* < 0.05)	Lachman test Laxity—KT-2000	IKDC HSS	Thomas splint and a Pearson attachment	√ (TTDPM)
			30°	1.34 ± 0.84°	30°	0.43 ± 0.37°	0.010								
			45°	1.72 ± 1.01°	45°	0.75 ± 0.52°	0.056								

ACLR, anterior cruciate ligament reconstruction; HSS, Hospital for Special Surgery Score; IKDC, International Knee Documentation Committee; NR, not reported; R, remnant preservation technique; RAP, reproduction of active positioning; RPP, reproduction of passive positioning; S, standard reconstruction technique

##### JPS test—RAP

Only one study [5] tested RAP before and after surgery with a mean follow-up duration of 7 months. The test results showed greater improvement of proprioception in ACLR-R compared with that in ACLR-S (*P* < 0.05) (Table 4).

##### JPS test—recording and testing

Three studies [11, 19, 28] recorded and compared the mean JPS value (test angle minus setting angle) of the reconstructed knees, while one study [5] recorded the inaccuracy of both legs (involved and contralateral normal knees) and reported side-to-side differences in the JPS value. Two studies used the Biodex system to measure JPS [5, 19], one study [28] used Thomas splint and Pearson attachment, while the remaining study [11] did not report on the testing apparatus. Only one study [19] described the test speed (with a speed of 5°/s) (Table 4).

##### TTDPM test

One study [28] measured TTDPM by continuous passive motion at final follow-up. Patients were tested at three angles of knee flexion with a speed of 0.5°/s. There was no statistically significant difference between ACLR-S and ACLR-R; however, the ACLR-R group showed better results at all angles (Table 5).

**Table 5:** Results of proprioception: threshold to detect passive motion

First author	Speed (°/s)	Direction (°)	TTDPM ACLR-S		TTDPM ACLR-R		*P* value	TTDPM measured knee	TTDPM measured times	TTDPM recorded value	Proprioception outcome measurements	Eye shades/earplugs
Lee et al. [25]	0.5	TE, 15, 30, 45	15°	1.33 ± 1.10°	15°	0.71 ± 0.62°	0.066	Injured and healthy knee	5	Mean value	Continuous passive motion	√
			30°	1.60 ± 0.87°	30°	1.18 ± 0.79°	0.975					
			45°	1.86 ± 1.30°	45°	1.30 ± 1.19°	0.617					

ACLR, anterior cruciate ligament reconstruction; R, remnant preservation technique; S, standard reconstruction technique; TE, toward extension

#### Balance tests, knee stability, and patient-reported outcomes

Only one study [28] reported on balance or postural tests. They conducted the one-leg hop test and single-limb standing test and found a statistically significant difference between the two groups (*P* < 0.05). Regarding knee stability and patient-reported outcomes, only one study [5] reported significantly greater improvement in anterior laxity tested by Rolimeter after ACLR-R compared with ACLR-S (*P* < 0.0001). None of the remaining studies found a significant difference (Table 4).

## Discussion

The most important observation of this review was that patients with ACLR-R showed improved postoperative proprioceptive evaluation results compared with those of the non-remnant ACLR-S. However, the long-term improvement of proprioception in ACLR-R remains unclear since the majority of studies failed to report long-term (> 16 months) follow-up results. Additionally, the heterogeneity of the characteristics and proprioceptive assessment of the studies prevented us from statistically evaluating the clinical outcomes.

Currently, there have been several meta-analyses or systematic reviews debating whether ACL tibial remnants should be saved during surgery [20, 25, 33, 34, 47, 48, 50]. Such reviews reported equivalent or superior postoperative clinical outcomes with ACLR-R compared with ACLR-S; however, there is insufficient scientific evidence supporting a definite conclusion. Moreover, these reviews [20, 25, 33, 34, 47, 48, 50] mainly concentrated on graft healing, synovial coverage, revascularization and ligamentization, knee stability function, and patient-reported outcomes, with a limited focus on proprioception or proprioceptive assessment. Therefore, our current review aimed to fill that gap by focusing on proprioception improvement.

Histological animal studies proved that ACL remnant preservation promoted new ingrowth of proprioceptors, neural cells, and nerve-related gene expression 6–12 weeks after surgery [23, 31, 45, 52], indicating the enhancement of proprioception of the knee joints in the early stage. The histological findings partially explained the results of our review, which reported a greater proprioceptive improvement in ACLR-R (compared with ACLR-S) in the short follow-up (≤ 12 months) period. Although there were a few findings of studies with longer follow-up that reported similar results, they lacked statistical significance [19, 28]. Histological studies in humans showed a reduction in the concentration of neural analogs in ACL grafts years after ACLR, regardless of graft source (allograft or autograft) [53]. Moreover, the effect of graft source on proprioceptive recovery has been unclear in several studies [7, 10, 39, 40] that have reported similar outcomes from ACLR with autograft, allograft, and artificial synthesis grafts. These results jointly indicate the potential benefits of remnant-sparing ACLR over the tensioning technique, and further comparisons of two techniques with different follow-up durations and graft sources in proprioception assessment and clinical outcomes are required in future studies.

Several human studies have evaluated the remnant-preserving effect after surgery with respect to remnant volume and surgical timing [29, 35, 44, 47]. However, the optimal volume and timing (time between the injury and the surgical procedure) for remnant-preserving ACLR in clinical practice require further investigation since only few studies reported the results of proprioceptive assessment. Only one study [19] (of those included in this review) described the mean time from injury to surgery. The varied descriptions of remnant volume in three included studies [5, 19, 28] also prevented us from performing subgroup analysis of the relationship between the remnant amount and proprioceptive restoration. Therefore, the effect of remnant volume and surgical timing during ACLR-R on proprioceptive recovery should be further studied.

Proprioception in this current review was mainly assessed with JPS (position sense) and TTDPM (movement sense). JPS is relatively easy to perform [37]. All studies included in the review reported on JPS [5, 11, 19], while only one study reported on TTDPM [28]. However, the two tests are commonly used for proprioception assessment, and both should be interpreted cautiously owing to the complexity of proprioception [37]. Furthermore, proprioceptors in the ACL and surrounding capsules and muscles [28] cannot be differentiated by any existing tests during assessment; thus, although JPS and TTDPM provided valuable information about joint position and movement sense, new tests are still needed for further investigation.

## Limitations

This study has few limitations. First, only four studies (level of evidence II or III) were finally extracted and analyzed in the review, and heterogeneity in study characteristics and outcome measures was encountered. Thus, the results were qualitatively summarized. Therefore, high-quality studies with validated outcomes are required in the future. Second, studies that used ACL augmentation with selective ACL anteromedial or posterolateral bundle reconstruction were excluded from the review to reduce the risk of bias between ACL reconstruction and augmentation. Further studies with respect to the different remnant-preserving ACLR techniques are needed for further investigation. Third, publication bias might have existed because only online-published English-language articles were included.

## Conclusion

The potential and benefits of remnant-preserving ACLR are apparent since improved results were observed in postoperative proprioceptive evaluation compared with the non-remnant standard ACLR.

More high-quality studies with validated tests are required to distinguish the effect of remnant preservation on knee proprioceptive restoration owing to the heterogeneity of existing studies.

## Data Availability

Not applicable.

## Abbreviations

ACL: Anterior cruciate ligament
ACLR: Anterior cruciate ligament reconstruction
ACLR-R: Anterior cruciate ligament tibial remnant-preserving reconstruction
ACLR-S: Standard technique
AM: Anteromedial
AAM: Accessory anteromedial
HSS: Hospital for Special Surgery
IKDC: International Knee Documentation Committee
JPS: Joint position sense
RAP: Reproduction of active positioning
RPP: Reproduction of passive positioning
TTDPM: Threshold to detect passive motion
